# Variation in sequences containing microsatellite motifs in the perennial biomass and forage grass, *Phalaris arundinacea* (Poaceae)

**DOI:** 10.1186/s13104-016-1994-6

**Published:** 2016-03-22

**Authors:** Susanne Barth, Marta Jolanta Jankowska, Trevor Roland Hodkinson, Tia Vellani, Manfred Klaas

**Affiliations:** Teagasc Crops Environment and Land Use Programme, Oak Park Research Centre, Carlow, Ireland; Department of Botany, School of Natural Sciences, Trinity College Dublin, Dublin, Ireland

**Keywords:** Poaceae, Microsatellite markers, *Phalaris arundinacea*, Reed canary grass, SSR

## Abstract

Forty three microsatellite markers were developed for further genetic characterisation of a forage and biomass grass crop, for which genomic resources are currently scarce. The microsatellite markers were developed from a normalized EST-SSR library. All of the 43 markers gave a clear banding pattern on 3 % Metaphor agarose gels. Eight selected SSR markers were tested in detail for polymorphism across eleven DNA samples of large geographic distribution across Europe. The new set of 43 SSR markers will help future research to characterise the genetic structure and diversity of *Phalaris arundinacea,* with a potential to further understand its invasive character in North American wetlands, as well as aid in breeding work for desired biomass and forage traits. *P. arundinacea* is particularly valued in the northern latitude as a crop with high biomass potential, even more so on marginal lands.

## Introduction

Slight changes in the genetic code, such as single nucleotide polymorphisms (SNPs) and single sequence repeats (SSRs) can be directly linked to phenotype differences. Hence, the development and characterisation of novel genetic markers can be of great help to breeders. SSRs have been commonly applied in quantifying genetic variation and analysing the gene flow and parentage in plants [1]. Some recent applications also include hybrid identification [2]. Single sequence repeats are abundant in the genome, multi-allelic and polymorphic and often can be cross-amplified on related species [3]. Next-generation sequencing can provide large numbers of SSRs as demonstrated in this study and is even more useful once converted into routinely applicable genetic markers.

**Fig. 1:**
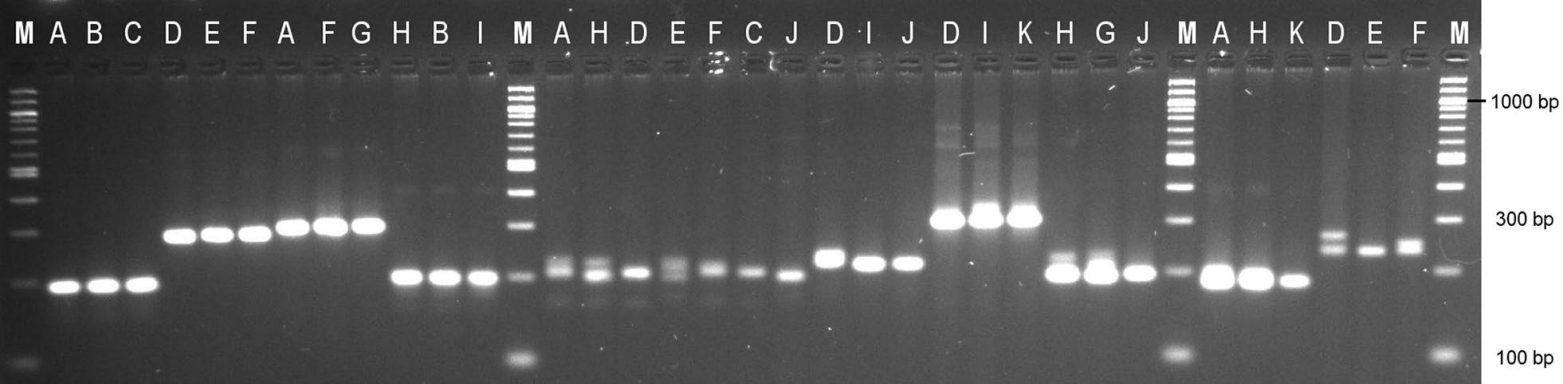
Representative image of amplified product for ten different EST-SSR markers. The samples were run on 3 % MetaPhor™ gel and three or, in one case seven different genotypes from wide geographic locations were amplified. The gel was post-stained with 3x gelRED for 1 h. *Lane* M—100 bp DNA ladder (New England BioLabs, Herts, United Kingdom); sample order and geographic distribution are defined in Table 1

The genus *Phalaris* belongs to the tribe Aveneae of the subfamily Pooideae of the grass and cereal family Poaceae [4]. Reed canarygrass (*Phalaris arundinaceaL*.) is a tall, perennial C3 grass which is distributed throughout Europe and in temperate regions of North America and Asia [5, 6]. On many sites it forms dense monospecific stands [7]. Considered an invasive wetland species [8, 9], *P. arundinacea* has been successfully introduced into nearly all continents except Antarctica. It is most commonly found growing along water margins and as such has been long recognised as a crop with a high biomass potential, particularly on marginal lands. Reed canarygrass, although not as productive as other grasses (i.e., *Panicum virgatum*) presents a unique set of characters that make it particularly tolerant to Northern climates [10]. Its high genetic variability has been observed in differences in production rates [8], forage yields [11] and photosynthetic characteristics [12] among others. Other non-crop uses of reed canarygrass include phytoremediation [13], erosion control [14] and paper production [15]. The newly developed primers presented in this publication were tested across a wide range of environmentally and climatically different regions from six European countries of Northern European distribution (Table 1). A subsample of three reactions per primer with one exception (primer TeaPh_nSSR_25) were chosen for sequencing (Fig. 1). These microsatellites can potentially be used for fingerprinting, GenBank accession characterisation and cross-amplification with other important and closely related forage grass species like *P. aquatica* [16]. Furthermore, microsatellite markers are routinely used to infer invasion routes of invading species [17], and as such could aid in understanding its invasive success in Northern America. Publically available genomic resources for the genus *Phalaris* are generally scarce; hence the primers are of high value for future research.

**Table 1:** Eleven genotypes characterized in this study

Country	Sample name	Latitude	Longitude
Poland	A	53° 50′02.83″N	21° 03′30.36″E
Poland	H	54° 23′21.42″N	18° 28′42.18″E
Germany	B	52° 13′09.87″N	11° 42′25.14″E
Germany	D	53° 25′51.60″N	09° 46′39.78″E
Denmark	I	56° 12′13.48″N	08° 09′39.07″E
Denmark	E	55° 56′16.98″N	12° 28′35.70″E
Sweden	F	58° 52′20.04″N	14° 53′56.79″E
Sweden	K	64° 36′27.31″N	20° 57′04.32″E
Ireland	G	53° 35′24.27″N	08° 03′33.69″W
UK	C	52° 29′17.12″N	00° 55′59.12″W
UK	J	52° 44′34.76″N	01° 08′09.87″W

The eleven genotypes are grouped by six European countries (sample names corresponding to Fig. 1) with latitude and longitude coordinates of their origin

## Methods

Total genomic DNA was extracted from eleven genotypes which were collected as part of the European Grass Margins project (Table 1) by either fresh extraction in liquid nitrogen or freeze-drying prior to extraction following a standard cetyltrimethylammonium bromide (CTAB) method [18]. Initially ninety primer pairs were designed using the Primer3 online programme (http://biotools.umassmed.edu/bioapps/primer3_www.cgi) from a normalized EST library consisting of 18,682 *P. arundinacea* transcripts [19] (sequence data available at the Sequence Read Archive at NCBI, accession number SRP045256) upon searching for microsatellites with Gramene SSRIT (Simple Sequence Repeat Identification Tool; http://archive.gramene.org/db/markers/ssrtool). The following core criteria were applied for primer design: (1) primer melting temperature between 57 and 63 ℃ with 60 ℃ as optimum; (2) primer size (bp) ranging from 18 to 27 ℃ with 20 ℃ as optimum; (3) product size (bp) ranging from 100 to a maximum of 400 with 200 as optimum; and (4) CG clump of two. We found microsatellites varying in repeat motifs from di- up to nona-, the majority of which were dinucleotide repeats. The primers used in this study were selected from a range of SSR repeat motifs (Table 2). All primers were synthesised by Metabion international AG and were subsequently tested by PCR on standard agarose gels first. Out of 90 initially tested SSR markers 43 were retained since they produced clear banding pattern in the expected size range on a 3 % MetaPhor™ agarose gel in 1 × TAE buffer. The further selection process of eight SSR markers which were characterized in detail was based on the indication that they might be highly polymorphic. The PCR products were run on a pre-stained gel with Ethidium bromide and placed at 4 ℃ for around 20 min to aid in obtaining optimal resolution and gel handling characteristics, as per manufacturer’s specifications. Primer details and GenBank accessions are provided in Table 3. A template DNA volume of 1 µL (40 ng/µL) was amplified with initial denaturation step for 5 min at 95 ℃ followed by 35 cycles each with a denaturation of 30 s at 95 ℃, 20 s at a primer specific annealing temperature, and extension of 20 s at 72 ℃, followed by a final extension at 72 ℃ for 7 min. The reaction mixture contained 1 × reaction buffer consisting of 1.25 µM dNTPs, 10 µM of each primer, and 0.5 U of Taq DNA polymerase (New England BioLabs, Herts, United Kingdom). The primers were tested for polymorphism originating from six European countries, in a total of eleven samples each from a different geographic region (Table 1). Purified PCR products (QIAquick PCR purification kit, Germantown, USA) were then sequenced by Sanger sequencing from both ends by GATC Biotech Ltd., London, England.

**Table 2:** A collection of forty three successfully amplified primers

Primer name	Isotiq position	Repeat motif	Primer sequence (5′-3′)	T_a_
TeaPh_nSSR_1	00060-1	(AC)_4_	F: TACTTCATTGGGTGGGATGG R: CGCGAATGAAATGAGAAAGC	54
TeaPh_nSSR_2	00060-2	(AT)_4_	F: GGTGGCTAATCTCAGGAATGG R: TGCCCGATAATAAGCACTAGC	54
TeaPh_nSSR_3	08186-1	(TTG)_4_	F: GGTAAATTCAGATTATTCCAAAACC R: CCTTTTTGAATGGCAGTTCC	53
TeaPh_nSSR_4	01314-2	(TGT)_4_	F: AACGGTGACAAAAGACAAAGC R: CAGCCGTATATCCACAATGC	54
TeaPh_nSSR_5	01313-2	(TGT)_4_	F: AACGGTGACAAAAGACAAAGC R: CAGCCGTATATCCACAATGC	54
TeaPh_nSSR_6	08185-3	(ATT)_4_	F: TGGCCAACTCTCAGTAGAAGG R: CCATGACCAAAATGAACTCC	53
TeaPh_nSSR_7	00075-2	(TTCT)_4_	F: TCCCCTCTTTGTTTATCATTCG R: GAATCCGGTAAGGTACTTTTGG	54
TeaPh_nSSR_8	00074-2	(TTCT)_4_	F: TCCCCTCTTTGTTTATCATTCG R: GAATCCGGTAAGGTACTTTTGG	54
TeaPh_nSSR_9	00068-2	(TTCT)_4_	F: TCCCCTCTTTGTTTATCATTCG R: GAATCCGGTAAGGTACTTTTGG	54
TeaPh_nSSR_10	03441-1	(TA)_4_	F: TGCAATGATTTTCTCTATCTTGC R: TCTATCGCTTCACTTTGTCTCG	53
TeaPh_nSSR_11	03440-1	(TA)_4_	F: TGCAATGATTTTCTCTATCTTGC R: TCTATCGCTTCACTTTGTCTCG	53
TeaPh_nSSR_13	00265-3	(GA)_4_	F: AGCAAGTATGCCGAAAGACC R: GGGAGACCCACACTTACAGC	54
TeaPh_nSSR_16	03439-1	(CT)_4_	F: GTACCCGAAACCGACACAGG R: CCCCCATACATGGTCTTACG	55
TeaPh_nSSR_17	03438-1	(CT)_4_	F: TTCTCCACGAGGCTCATACC R: GAAGTTACGGGGCTATTTTGC	55
TeaPh_nSSR_19	03440-4	(AT)_4_	F: TTCCGAATTAAATGGAGAATCC R: GATAACGGGACATGAAGACTCC	54
TeaPh_nSSR_20	00072-1	(AT)_4_	F: GGTGGCTAATCTCAGGAATGG R: TGCCCGATAATAAGCACTAGC	54
TeaPh_nSSR_21	08188-1	(AG)_4_	F: CAATGCCAAAGAAACAATGC R: ACCTCAGATCGAAGCATTCC	54
TeaPh_nSSR_23	00071-1	(AC)_4_	F: TACTTCATTGGGTGGGATGG R: CGCGAATGAAATGAGAAAGC	56
TeaPh_nSSR_24	03440-3	(AT)_5_	F: GAATGAAAATGCCAATAAAGTCG R: TTTTATTTCTCTAATTCGCAAATCC	54
TeaPh_nSSR_25	08351-5	(TGC)_10_	F: TCCTATGATCTCTGCCTCAGC R: GCACTGTCCATCAACACACC	55
TeaPh_nSSR_34	01672-8	(CCGAAACA)_3_	F: TTACCGACTCCGTCTTGACC R: GTCGATGGAGATGACGTTGG	55
TeaPh_nSSR_37	03471-10	(TTTTGAA)_3_	F: GTGTTTGGCCTGTAATCTGG R: CGTAAATGCATCTCTATCTGTTCC	53
TeaPh_nSSR_42	01705-4	(GT)_8_	F: TCAAGTGTCATCCGTTGTCC R: TTTTAACGCAAATAGTTTCATCG	53
TeaPh_nSSR_43	02516-1	(GT)_6_	F: TGGACTGCACCTAGGAGACC R: TACCACCATGGAACAAAACG	54
TeaPh_nSSR_45	08327-5	(GC)_4_	F: AAAGTACATTGAAAGCTAGTGTCACC R: GCCTCCAAAGCAAGATGC	54
TeaPh_nSSR_46	03588-3	(CG)_6_	F: TCTCCGCTCGATCTAAATAGC R: TGTGTGTGCTGAAAGTGTCG	55
TeaPh_nSSR_47	01700-4	(CG)_4_	F: GACAGATGGGGCACTACTCC R: GTGTGAGGAATCCACAGTGC	54
TeaPh_nSSR_48	02594-3	(TAA)_6_	F: AAGAGTGTCACCATGGAGTGG R: ACCTTCTGAGAGCCTCTTGC	54
TeaPh_nSSR_49	02597-9	(GCA)_5_	F: GATACGCTGGAATACCAGAAGG R: GGGAATGGAAACGAACAGG	55
TeaPh_nSSR_50	08302-4	(GAA)_5_	F: AAGAGGAAGCCGAAGAGTGG R: TCTGTGGTGCTCAGTTCAGG	55
TeaPh_nSSR_52	08189-10	(GAT)_3_	F: TTAACTCGAGGTCATGCATCC R: CCTTTAGCGTCCAAAACTGC	55
TeaPh_nSSR_54	08427-4	(CAA)_4_	F: ACATCCACAGGATTCCATGC R: GCCAGAGATGAGAAGGATGC	55
TeaPh_nSSR_55	02553-8	(CAT)_3_	F: AGCAACCAGAACCTGACACG R: AGATGGTACGGCTGGTATGC	55
TeaPh_nSSR_57	02609-7	(CGG)_4_	F: GTTCGCTTCGATTTGTTTCC R: CGAAATGAACGGCCTAATCC	55
TeaPh_nSSR_58	08459-13	(GCTC)_4_	F: TCCCGACTTCATGAGCTACC R: GGAGGAGCATGTGTGAATGG	55
TeaPh_nSSR_59	00075-2	(TTCT)_4_	F: TCCCCTCTTTGTTTATCATTCG R: GAATCCGGTAAGGTACTTTTGG	54
TeaPh_nSSR_60	01318-11	(AGGA)_3_	F: GGGCTTTCTACATAGGGATCG R: TTGATCTTTACGGTGCTTTCC	54
TeaPh_nSSR_65	08352-6	(AGG)_4_	F: CTCCACCACCTCCACAAAAT R: TTTCGTCTTTGTGCTTGCTG	55
TeaPh_nSSR_66	00769-7	(TTG)_3_	F: CGTTGTGCCTTAGCTACTTGC R: ATGATCCAACCAGCTTGACC	55
TeaPh_nSSR_70	08235-11	(TGCT)_3_	F: CCTTGAGGAGGATGATGTGG R: TCCTGATGTGCTTGATGAGC	55
TeaPh_nSSR_71	08212-11	(TTCA)_3_	F: GATGGAATCACGCTCTGTAGG R: GGGCAGTAGCGAAGAGATCC	55
TeaPh_nSSR_80	03674-1	(GAT)_4_	F: CCAAACCCAGTTGTGACTCC R: GGCATCAGAATCATAGTCATCG	55
TeaPh_nSSR_81	03659-2	(GGT)_5_	F: CGGTTGGACTGATAACATTGG R: CCCATCCTGAGTCGTCACC	55

Forty three primers that successfully amplified a distinct band of expected size on a 3 % MetaPhor agarose gel, their Isotiq position on the EST assembly, repeat motif, forward and reverse primer sequences (5′-3′) and annealing temperature (Ta)

**Table 3:** Eight highly polymorphic nuclear EST-SSR markers with GenBank accession numbers and size range

Primer name	Clone; GenBank accession no.	Size range (bp)
TeaPh_nSSR_2	KU316389; KU316392; KU316395	191
TeaPh_nSSR_4	KU316399; KU316400; KU316403; KU316416; KU316414	240–241
TeaPh_nSSR_7	KU316423; KU316427; KU316426; KU316429; KU316433	247
TeaPh_nSSR_24	KU316439; KU316441; KU316443; KU316444	197
TeaPh_nSSR_46	KU316451; KU316452; KU316458; KU316467; KU316471	178–186
TeaPh_nSSR_47	KU316485; KU316486; KU316489; KU316476	261–269
TeaPh_nSSR_49	KU316491; KU316492; KU316495; KU316498	152–166
TeaPh_nSSR_80	KU316500; KU316501; KU316506; KU316512	144–150

The study developed eight polymorphic EST-SSR markers containing polymorphism such as SSRs, InDels and SNPs

## Results and discussion

**Fig. 2:**
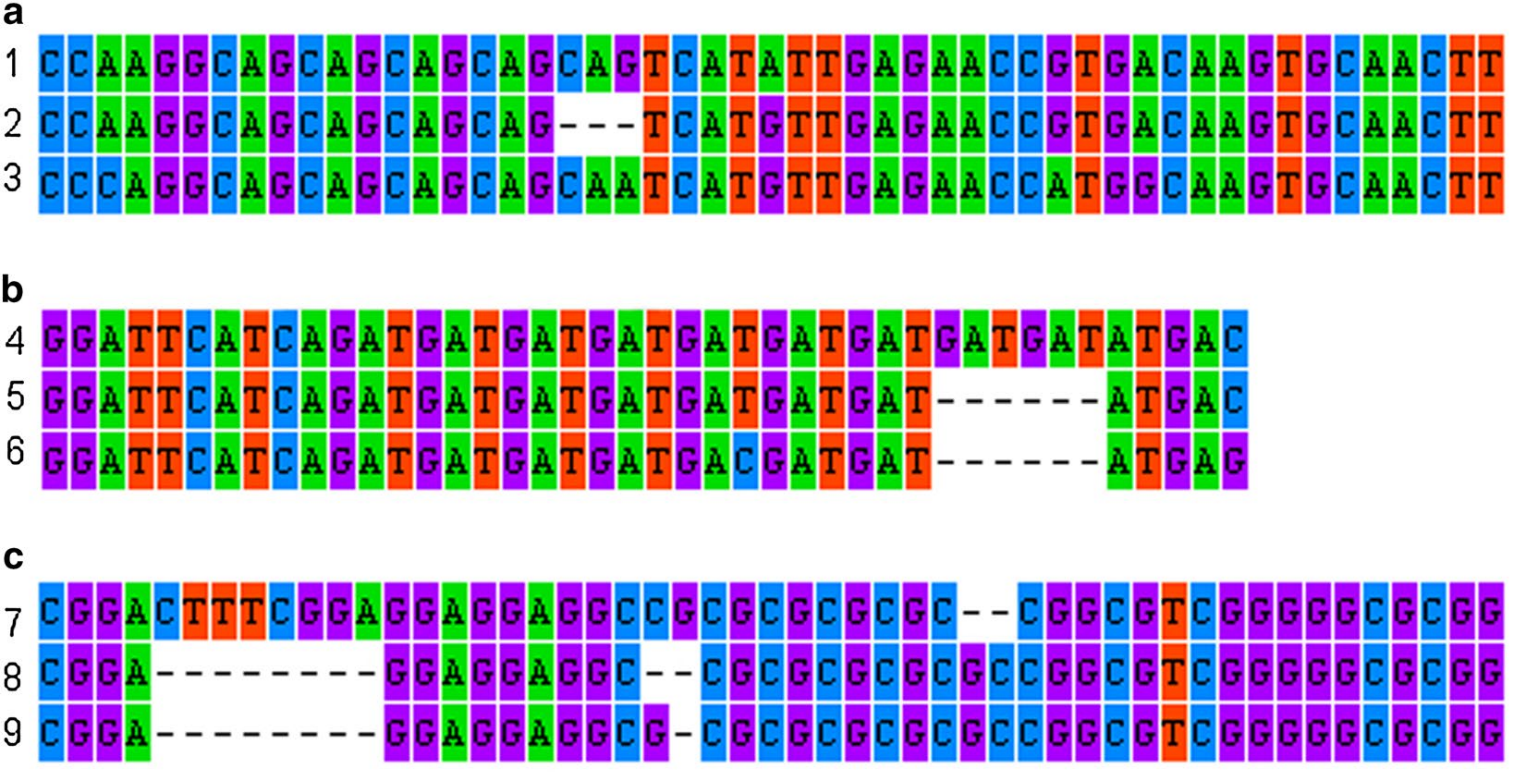
Example of motif changes, indels and SNPs in the microsatellite region of nine representative samples. **a** fragments amplified with primer TeaPh_nSSR49; *1* Poland (accession number KU316491); *2* Ireland (accession number KU316495); *3* Ireland (accession number KU316498).** b** fragments amplified with primer TeaPh_nSSR80; *4* Poland (accession number KU316501); *5* Poland (KU316502); *6* Poland (KU316514). **c** fragments amplified with primer TeaPh_nSSR46; *7* Germany (accession number KU316457); *8* Denmark (KU316467); *9* United Kingdom (KU316471)

43 out of the 90 initially designed microsatellite marker primer pairs proved to amplify successfully with discreet bands. Eight highly polymorphic SSR loci for *P. arundinacea* were identified which ranged in size from around 300 to 200 bp in length (Table 3). Sanger sequencing revealed numerous single nucleotide polymorphisms (SNPs), motif changes as well as indels in the genotypes of *Phalaris* from the different geographical locations (Fig. 2). Changes in the motif length varied from tetra- to tri-, di- and even mononucleotide repeats. In some instances the repeat motif was longer than expected (from tetra- to hepta- and nonanucleotide repeats). Changes were also observed from penta- to tetra- and hexanucleotide repeats. These microsatellite markers are useful for studying genetic diversity and population structure as well as for elucidation of *P. arundinacea* invasive status. As an increasingly important energy crop and well established forage crop species, the improvement of bioenergy and palatability traits for livestock, *P. arundinacea* might be of interest to breeders worldwide. The markers can be also further cross-amplified in closely related taxa like *P. aquatica*, an important forage species in Australasia, and other members of the genus *Phalaris* like *P. minor* a widely spread grass weed. These eight microsatellite markers will be of interest and value in addressing taxonomic and biogeographic issues because of the samples’ wide geographic distribution.
